# Hip prosthesis and colon surgery, a decade of surveillance on surgical site infections in Italy, a prospective cohort study: rates, trends, and disease burden in DALYs

**DOI:** 10.1186/s13756-024-01377-6

**Published:** 2024-02-12

**Authors:** Valerio Bordino, Costanza Vicentini, Alessandro Roberto Cornio, Maria Michela Gianino, Carla Maria Zotti

**Affiliations:** Paediatrics and Public Health Sciences Department, University of Turin, Via Santena 5bis, 10126 Turin, Italy

**Keywords:** Surveillance programs, Surgical site infections (SSIs), Hip arthroplasty, Colon surgery

## Abstract

**Background:**

Surveillance programs are a key element of interventions aiming to reduce rates of surgical site infections (SSIs). The aim of this study was to evaluate rates and trends of SSIs following hip arthroplasty and colon surgery procedures in Piedmont, a region in North-western Italy, from 2010 to 2019. Further, we aimed to assess the burden of SSIs in terms of Disability-Adjusted Life-Years (DALYs).

**Methods:**

A prospective cohort study was conducted among 42 hospitals participating in the surveillance system. Procedure-specific SSI rates were calculated and the 2010 - 2019 trend was evaluated using Spearman's Rho test. Patients were stratified according to age, sex and infection risk index according to life expectancy in order to calculate DALYs, using a modified version of the ECDC’s BCoDE toolkit: disease models for both procedure types were adapted to incorporate long-term disability associated with SSIs.

**Results:**

Overall, 20,356 hip arthroplasty and 11,011 colon surgery procedures were monitored over 10 years and were included in our analyses. Hip arthroplasty and colon surgery cumulative SSIs rates were 1.5% and 8% respectively. Using the Spearman’s Rho test, we evaluated a significant downward trend from 2010 to 2019 for colon surgery interventions (Rs − 0.7, *p* < 0.05), while there was no difference for hip arthroplasty. (Rs − 0.04, *p* > 0.05).

Regarding disease burden, a total of 955.3 (95%CI 837.7–1102.98) and 208.65 (95%CI 180.87–240.90) DALYs were calculated for SSIs following hip arthroplasty, whilst 76.58 (95%CI 67.15–90.71) and 38.62 (95% CI 33.09–45.36) DALYs for SSIs in colon surgery, in 2010 and 2019, respectively.

**Conclusions:**

The significant decrease both in terms of incidence and DALYs found in this study for colon surgery and the stability for hip arthroplasty support the role of surveillance networks in improving quality of care. Despite the smaller SSI rate, the burden associated with SSIs following hip arthroplasty was higher, which is important to consider in light of the aging population in Italy.

**Supplementary Information:**

The online version contains supplementary material available at 10.1186/s13756-024-01377-6.

## Background

Healthcare-associated infections (HAIs) are a major public health issue affecting all care settings, with an estimated 1 in 31 hospitalized patients acquiring at least one infection during their stay in the United States [1]. HAIs can result in increased morbidity, mortality, and healthcare costs, and surgical site infections (SSIs) are among the most common and costly HAI types [2]. SSIs are defined as infections occurring at or near a surgical incision within 30 days of surgery, or within one year if an implant is left in place [3].

In Italy the prevalence of healthcare-associated infections was estimated to be 7.26% according to the ECDC point prevalence study in 2016 [4]. Hospitals, which are traditionally perceived as places of healing, have also become known as potential sources of infection and exacerbation of pre-existing conditions [5].

In response to the growing recognition of the risks posed by HAIs, numerous IPC strategies have been developed. National and local policies have aimed to reduce hospitalization times and implement measures to enhance hand hygiene practices and staff training [6]. Structural measures have been put in place to allow the isolation of patients with severe comorbidities and to increase the flexibility of hospitals to adapt to new challenges [5].

Within the landscape of HAIs, SSIs are particularly relevant due to their frequency, potential severity, and associated costs. SSIs are associated with longer post operative hospital stays, increased morbidity and mortality, and may require additional surgical procedures or intensive care [3]. The incidence of SSIs varies widely depending on the type of surgery performed, with some estimates suggesting that up to 20% of patients undergoing certain types of surgeries may develop SSIs [3].

Numerous strategies have been studied to limit the onset of SSIs, including pre- and peri-operative interventions such as antibiotic prophylaxis and wound management techniques [3, 7]. The use of bundled interventions, in which multiple interventions are implemented in combination, has proven to be particularly effective in reducing SSI rates [8]. Surveillance systems are necessary to monitor trends in SSI rates and to evaluate the effectiveness of prevention strategies [2], and are a core component of IPC efforts.

In 2008, the region of Piedmont joined the Italian national surveillance system (Sistema Nazionale Sorveglianza Infezioni del Sito Surgico [SNICh]) for SSIs, which, founded in 2006, is coordinated by the Emilia-Romagna Regional Health and Social Agency (ASSR) and based on The European Surveillance System (TESSy) for communicable diseases [9]. The surveillance system has maintained its activities since then [10]. Even during the COVID-19 pandemic, Piedmont's hospitals were asked to monitor a minimum number of procedures.

In a previous Italian study, based on national data from a Point Prevalence Study, the burden of disease in terms of Disability-Adjusted Life-Years (DALYs) due to SSIs was estimated for 2016 to be 36,429 DALYs, covering a 9% of the total Italian burden of HAIs [11].

The aim of this study was to evaluate the rates and trends of SSIs following hip arthroplasty and colon surgery in Piedmont from 2010 to 2019, as these are the most closely monitored procedures in the region. Additionally, we aimed to provide a quantitative assessment of the disease burden related to SSIs in these surgical groups, as measured by DALYs.

## Methods

### Data collection and analysis

All public acute-care Piedmontese hospitals participate in the s urveillance system. ata were collected through SNICh s, following the European Center for Disease Prevention and Control (ECDC) 2016 v2.0 Protocol [9]. Data were continuously collected from 2010 to 2019, and all hip arthroplasty and colon surgery procedures, according to the ECDC protocol, were monitored during this period were considered, including those that resulted in at least one SSI. Surveillance was carried out by the surveillance team, made up of a doctor from the health management and infectious risk specialized nurses. Post-discharge surveillance timing is 30 days. When prosthetic material is implanted, the timing is 90 days. Both elective and emergency procedures were included. Procedures are categorized using the infection risk index (IRI), which is calculated following NHSN methodology, according to: procedure duration, the patient’s American Society of Anaesthesiology (ASA) score and wound contamination class (clean, clean-contaminated, contaminated, dirty). The methodology for data collection through SNICh in Piedmont was previously described in detail [10]. A web based platform built by University of Turin was used to collect Regional data.

A four-element bundled intervention was introduced in hospitals in Piedmont participating in the SSI surveillance network as part of the regional performance indicator system. Hospitals were invited to participate in the intervention on a voluntary basis. In total, 29 hospitals implemented the bundle out of the 49 hospitals of the region of Piedmont participating in SNICh. The four elements of the bundle are: preoperative showering, appropriate hair removal, antimicrobial prophylaxis, maintenance of intraoperative normothermia.

Procedure-specific, overall, and yearly SSI rates were calculated. Trend analyses for SSI rates were performed using Spearman’s Rho. Comparisons between 2010 vs 2019 DALYs for SSIs following colon and hip surgery were estimated using χ^2^ test. Statistical analysis was conducted using IBM SPSS Statistics v28.0 software.

### DALY calculation

The burden of disease presented in DALYs was calculated for the years 2010 and 2019 for SSIs following both procedure categories. The Burden of Communicable Disease in Europe (BCoDE) toolkit, v2.0, provided by the ECDC, was used for the construction of procedure-specific outcome trees and for the calculation of DALYs [12]. Two specific outcome trees were created using literature data to account for disabilities associated with SSIs following colon and hip surgery [11, 13].

We carried out a literature search for HPRO progression probability and disability weights (DW) [13–15] and for colon surgery progression probability and its corresponding DW [14, 16].

The death probability following SSI was extrapolated from SNICh [10].

DALY is a composite health gap measure that includes the years of life lost (YLL) with the years lost due to disability (YLD) [17].

The methodology for DALY calculation has been previously described [11]. This study is based on SSI regional incidence data to calculate DALYs. Based on the population of the SNICh program, with projections on hospital discharges following hip arthroplasty and colon surgery in Piedmont, the population was divided by age, sex, and life expectancy. Life expectancy was approximated using the ASA score as a proxy for McCabe score, using the same methodology as Koek et al. [14] After the process of creating the outcome trees and inputting the study population data projected on the regional population, 10,000 Monte Carlo iterations were run.

### Ethical aspects

Considering the program’s purposes are disease surveillance and improvement of quality of care, and that the program is coordinated by public entities (Italian Centre for Disease Control, CCM, Italian Ministry of Health, Regions of Emilia-Romagna and Piedmont), the SNICh protocol states that the written consent of involved patients or any other authorization from Ethics Committees or the Protection Commissioner is not requested. Patients are notified of their participation in the program via an information sheet and only anonymized data are collected.

## Results

Over the course of the 10-year study period, a growing number of wards participated in the surveillance for the two groups of interventions assessed, with a total of 11,417 supervised interventions for colon surgery and 20,804 for hip arthroplasty surgery (as shown in Tables 1, 2, 3). The tables provide information on the number of participating hospitals, monitored interventions, and SSIs (stratified by year of surveillance) for both colon surgery (Table 2) and hip arthroplasty (Table 3).

**Table 1:** Study population 2010 and 2019

	Colon Surgery 2010	Colon Surgery 2019	Hip arthroplasty 2010	Hip arthroplasty 2019	Colon surgery 10 years overall	Hip arthroplasty 10 years overall
Male N (%)	428 (51%)	820 (55%)	120 (42%)	1350 (41%)	6073 (53%)	8063 (39%)
Age median (IQR)	72 (64–79)	72 (61–80)	70 (60–77)	74 (65–81)	72 (63–80)	75 (66–82)
ASA score 1 N	37 (4%)	103 (7%)	24 (8%)	286 (9%)	734 (6%)	1801(9%)
ASA score 2 N	375 (45%)	606 (41%)	136 (47%)	1768 (54%)	5260 (46%)	10,499 (50%)
ASA score 3 N	298 (36%)	599 (40%)	79 (27%)	1085 (33%)	4450 (39%)	7339 (35%)
ASA score 4 N	62 (7%)	153 (10%)	4 (1%)	109 (3%)	899 (8%)	717 (3%)
ASA score 5 N	6 (1%)	11 (1%)	0 (0%)	1 (0%)	65 (1%)	16 (0%)
ASA score unknown	45 (5%)	18 (1%)	45 (16%)	46 (1%)	262 (2%)	600 (3%)
Wound class 1	28 (3%)	16 (1%)	282 (98%)	3281 (99%)	230 (2%)	20,689 (99%)
Wound class 2	480 (57%)	1103 (74%)	6 (2%)	11 (1%)	7962 (70%)	72 (1%)
Wound class 3	227 (27%)	271 (18%)	0 (0%)	2 (0%)	2233 (20%)	19 (0%)
Wound class 4	80 (10%	98 (7%)	0 (0%)	0 (0%)	965 (8%)	3 (0%)
Wound class unknown	21 (3%)	2 (0%)	0 (0%)	0 (0%)	27 (0%)	21 (0%)
TOTAL	836	1490	288	3294	11,417	20,804

Table shows study population data: Sex, Age, Asa score for Colon and hip arthroplasty 2010 and 2019. *IQR* inter quartile range, *ASA* American Society of Anesthesiologists (ASA) physical status classification system

**Table 2:** SSIs following colon surgery

Year	N. OF SSIs	N. of monitored procedures	% SSI (%)	Patients with an SSI, median (IQR)	N. of participant hospitals
2010	77	836	9.21	72 (66–78)	23
2011	40	394	10.15	74 (63–80)	14
2012	152	1326	11.47	71 (64–79)	27
2013	96	1251	7.68	71 (61–78)	26
2014	101	1070	9.44	74 (62–81)	18
2015	111	1082	10.26	70 (63–78)	18
2016	72	1153	6.25	71 (62–79)	19
2017	83	1120	7.41	73 (65–79)	19
2018	119	1695	7.03	70 (61–79)	24
2019	85	1490	5.70	74 (63–79)	22
TOTAL	936	11,417	8.19	71 (63–79)	

The table shows the number of Surgical Site Infections (SSIs) following colon surgery that were monitored from 2010 to 2019, along with the number of surveilled procedures and participant hospitals in each year. The total number of SSIs, surveilled procedures, and participant hospitals are also shown at the bottom of the table. *SSIs* surgical site infections. *IQR* inter quartile range

**Table 3:** SSIs following hip arthroplasty

Year	N. of SSIs	N. of monitored procedures	% SSI (%)	Age of patients with an SSI, median (IQR)	N. of participant hospitals
2010	3	288	1.04	63 (62–69)	6
2011	5	386	1.30	72 (51–79)	7
2012	59	2046	2.88	74 (67–83)	28
2013	38	2232	1.70	74 (69–82)	21
2014	29	2469	1.17	78 (74–83)	25
2015	21	2296	0.92	76 (72–83)	19
2016	31	2302	1.35	79 (73–86)	20
2017	34	2389	1.42	73 (58–79)	18
2018	31	3102	1.00	77 (68–82)	22
2019	46	3294	1.40	77 (70–82)	22
TOTAL	297	20,804	1.43	76 (69–83)	

Incidence of surgical site infections (SSIs) following hip arthroplasty surgery in participating hospitals in Italy from 2010 to 2019. The table shows the number of SSIs, the number of surveilled procedures, and the number of participating hospitals for each year. The total number of SSIs and surveilled procedures over the 10-year period is also shown

*SSIs* surgical site infections. *IQR* inter quartile range

Using the Spearman’s Rho test, we evaluated a significant downward trend from 2010 to 2019 for colon surgery interventions (Rs − 0.7, *p* < 0.05), while there was no difference for hip arthroplasty. (Rs − 0.04, *p* > 0.05).

Table 4 shows the burden of disease expressed in terms of DALYs for hip arthroplasty and colon surgery for the years 2010–2019, with the population divided into three groups based on life expectancy as determined by the McCabe score. As shown by Table 4 there was a clear reduction in the burden of disease for colon surgery over the 10-year period, with a significant difference (χ^2^ test *p* < 0,05), while for hip arthroplasty we note a slight increase in the burden (χ^2^ test *p* < 0.05).

**Table 4:** DALYs in 2010 and 2019 for Colon and Hip arthroplasty surgeries

Surgery	Year	Aggregate DALY per Year	Aggregate DALY per 100 SSI	McCabe1 DALY per Year	McCabe1 DALY per 100 SSI	McCabe2 DALY per Year	McCabe2 DALY per 100 SSI	McCabe3 DALY per Year	McCabe3 DALY per 100 SSI
COLON	2010	76.58	2.18	46.83	2.40	28.59	2.25	1.17	0.40
	IC95%	(67.15–90.71)	(1.85–2.56)	(40.59–54.06)	(2.06–2.82)	(24.49–33.58)	(1.97–2.66)	(1.02–1.36)	(0.35–0.47)
	2019	38.62	1.32	20.58	1.44	17.12	1.45	0.92	0.29
	IC95%	(33.09–45.36)	(1.12–1.52)	(17.63–23.96)	(1.26–1.68)	(14.49–19.93)	(1.23–1.72)	(0.80–1.06)	(0.25–0.33)
HPRO	2010	955.30	1.17	899.08	1.54	56.20	2.51	0.02	0.02
	IC95%	(837.70–1102.98)	(1.01–1.36)	(770.42–1065.05)	(1.32–1.78)	(49.28–66.57)	(2.12–2.89)	(0.01–0.02)	(0.018–0.023)
	2019	208.65	2.00	100.87	1.52	106.39	3.10	1.39	0.41
	IC95%	(180.87–240.90)	(1.70–2.37)	(87.44–117.47)	(1.33–1.75)	(93.29–122.83)	(2.65–3.57)	(1.19–1.63)	(0.36–0.48)

*DALY* disability-adjusted life year, *HPRO* hip arthroplasty surgery. McCabe classification is a method used to determine the severity of an underlying illness, with higher numbers indicating greater severity. The numbers in parentheses represent 95% confidence intervals

## Discussion

SSIs are associated with a significant burden of disease for patients and healthcare systems worldwide [2, 18]. In this study, we presented the trends in SSI incidence and burden of disease expressed in DALYs in Piedmont over a decade.

Our data showed a statistically significant decrease in SSI incidence over the 10-year period, leading to a notable reduction in the burden of disease expressed in DALYs when comparing the first and last years of the study.

We focused on two types of surgical procedures, hip arthroplasty and colon surgery, as they were the most monitored over the 10 years. Despite some peaks over the years, a decreasing trend in SSIs following colon surgery was evident, while in hip arthroplasty the incidence rate remained stable. Concerning DALYs, the difference between 2010 and 2019 was significant for both surgeries, with a reduction in colon surgery and a slight increase in hip arthroplasty. This small increase is most likely due to the McCabe 3 group, which is the only one who saw a significant increase in the number of DALYs. Surveillance data suggest there has been an increase in the number of operations performed on patients with serious comorbidities, who are therefore more prone to surgical site infections with a serious prognosis. esults of this study (see Additional files 1–4), suggest that the McCabe 1 group has a high incidence of cases, however these are associated with a low mortality. Conversely, i n the McCabe 3 group,, a lower incidence was found but cases were associated with a greater number of YLL. YLDs we e more represented in the McCabe 1 group, as a person with few comorbidities and a longer life expectancy acquiring disability would experience impairment for a longer period.

It is worth noting that despite hip arthroplasty surgeries having a lower SSI rate than colon surgery, the burden of SSIs following hip arthroplasty was higher in 2019. This could be attributed to the higher frequency of hip arthroplasty surgeries in elderly patients, who are more susceptible to long-term disability and functional impairment resulting from SSIs, particularly in the context of a surgery that is typically associated with higher levels of disability [25–27]. In Tables 1, 2, and 3, it’s noteworthy that patients undergoing hip arthroplasty tend to be older compared to those undergoing colon surgery, particularly in the latter years of this study. This demographic variance could potentially elucidate the higher burden observed in hip arthroplasty compared to colon surgery. Additionally, it’s evident that, on average, the ASA Score of patients undergoing hip arthroplasty is better than that of patients undergoing colon surgeries. Given the aging population in Italy and other developed countries, it is important to consider the long-term disability associated with SSIs in this patient population.

It is worth noting that the comparison of SSI rates between studies can be challenging due to differences in study design, patient populations, and data collection methods. For example, our study focused on a specific Italian region, while other studies may have included multiple regional settings or different patient populations. Nevertheless, our findings are consistent with previous studies that have reported a decline in SSI rates over time [10]. For instance, Magill et al. [19] conducted a multistate point-prevalence survey of healthcare-associated infections and found that SSI rates decreased from 2.1% in 2011 to 1.9% in 2012. Similarly, Leaper and colleagues (2004) estimated a decline in SSI rates in Europe from 7.1% in 1986 to 4.1% in 1997 [18].

Several IPC measures have been implemented in our region to reduce the incidence of SSIs, such as, the World Health Organization (WHO) global guidelines for the prevention of surgical site infections, which emphasize the importance of hand hygiene, antimicrobial stewardship, and surgical site preparation [20]. The implementation of IPC guidelines and the use of checklists during surgical procedures may have helped to prevent infections and improve patient outcomes. Bundle approaches combining multiple measures have also been developed and recommended in our region to enhance the effectiveness of IPC strategies [8, 21–24].

Findings of this study suggest that efforts to improve IPC practices and patient safety in surgical settings in our region may have had a positive impact on patient outcomes in terms of YLLs and YLDs. Nonetheless, determining the specific impact of IPC measures on SSI rates or burden was not the objective of this study.

The active participation of healthcare facilities is crucial in reducing the incidence of SSIs and the associated burden of disease [10].

The experience of our surveillance program, which monitored SSIs in numerous facilities over a period of over 10 years, demonstrates that consistent and standardized surveillance methods can yield significant results.

Despite the disruption due to the COVID-19 pandemic, participating hospitals were invited to pursue surveillance efforts and to continue monitoring even the limited number of urgent procedure s they were performing. This was done to maintain the surveillance protocol and provide essential continuity to the program. It will be interesting to evaluate the impact of COVID-19 on the burden on SSIs, with the addition of longer term data (e.g. impact on waiting lists and of delayed interventions).

The COVID-19 pandemic has highlighted the critical importance of early detection, containment, and mitigation of infectious diseases. Through the adoption of IPC measures and active participation in surveillance programs, healthcare facilities can help prevent the spread of infectious diseases, including SSIs. It is crucial to maintain continued adherence to these measures to maximize their effectiveness.

## Limits and strengths

Our study has some limitations that must be considered. First, the participation of healthcare facilities in the surveillance program was voluntary, even though the SNICH protocol requires a minimum of 50 consecutive interventions each year. The participating wards may have been more attentive to IPC measures and may not be representative of all wards of the single hospitals. Second, our study did not evaluate the impact of specific IPC measures on SSI incidence or burden of disease. Further, several confounding factors may have played an important role in the decrease of SSI, such as increased for example more focus on anastomotic leakage prevention, increased use of disposables, use of different suture material/stapler devices or other interventions. Third, our DALY calculations may be subject to some degree of uncertainty due to the use of disease models. In particular, our YLD estimations are based on incidence rather than prevalence data, as indicated by the GBD 2010 study [28].

However, we used this approach in order to allow comparison with previous research using our same methodology [14]. Further, the prevalence-based approach was proposed as both incidence and average duration of disease estimates are necessary for YLD calculations, whereas for several diseases only prevalence data are available [28]. In our case, we used primarily collected incidence data and retrieved from the literature disease duration estimates. In line with the 2010 GBD simplified DALY calculation approach, no time discounting nor age-weighting were applied to our calculation. Comparing DALYs of two time points has limitations for detecting trends in time and could be dependent on the year selected for analyses. Finally, we adjusted life expectancies based on ASA scores in order to account for comorbidities, as proposed by Koek et al.

Strengths of our study are the fact that few studies have assessed the burden of SSIs in terms of DALYs. A notable characteristic is that we built a personalized and literature-based outcome tree to estimate the DALYs associated with SSIs following hip arthroplasty and colon surgery procedures. The use of such a tool allowed us to take into account the long-term disability associated with SSIs, which is often not accounted for in previous studies. This approach could be beneficial in future studies to estimate the burden of other HAIs. Our study provides further evidence for the importance of dividing the population into three groups based on their life expectancy and comorbidities to assign YLLs and YLDs [11, 29].

A further advantage of this study is the utilization of a sizable dataset at the regional level.

In light of our findings and limitations, there are several areas for future research: the evaluation of other surgical procedures besides hip arthroplasty and colon surgery could provide a more comprehensive picture of the SSI burden in the region; it would be valuable to conduct similar surveillance studies in other regions or countries to assess the generalizability of our results and this would allow for a more comprehensive understanding of the burden of SSIs. Additionally, future studies could investigate the effectiveness of specific IPC measures, such as the use of surgical bundles or other strategies to reduce SSI incidence.

## Conclusions

In conclusion, our study showed, for colon surgery, a significant decrease in SSI incidence and burden of disease expressed in DALYs over 10 years in Piedmont, Italy. Our results showed a stable trend in both SSI incidence and DALYs for hip arthroplasty. Continuous surveillance and monitoring of SSIs using standardized methods and the active participation of healthcare facilities can yield important results. Our findings also demonstrate the significant burden of SSIs in terms of DALYs, particularly following hip arthroplasty procedures. Further studies evaluating the impact of specific IPC measures and the cost-effectiveness of surveillance programs are useful to enhance the effectiveness of SSI prevention and control strategies.

### Supplementary Information

**Additional file 1**. Characteristics of Supplement Files.

**Additional file 2**. Hip prosthesis 2019 aggregated daly - BCoDE Toolkit output. Bubble charts are shown comparing different disease models, in which the size of each bubble corresponds to the magnitude of the burden of disease expressed in DALYs per 100,000 population. In the first graph the x-axis represents the estimated incidence per 100,000, while the y-axis represents the estimated mortality per 100,000 population calculated through the disease model. The second graph differs only by showing DALYs per case on the y-axis. The “Aggregate results” page also shows age-group and sex-stratified tables and bar charts.

**Additional file 3**. Hip prosthesis 2010 aggregated daly. - BCoDE Toolkit output. Bubble charts are shown comparing different disease models, in which the size of each bubble corresponds to the magnitude of the burden of disease expressed in DALYs per 100,000 population. In the first graph the x-axis represents the estimated incidence per 100,000, while the y-axis represents the estimated mortality per 100,000 population calculated through the disease model. The second graph differs only by showing DALYs per case on the y-axis. The “Aggregate results” page also shows age-group and sex-stratified tables and bar charts.

**Additional file 4**. Colon surgery 2010 aggregated daly- BCoDE Toolkit output. Bubble charts are shown comparing different disease models, in which the size of each bubble corresponds to the magnitude of the burden of disease expressed in DALYs per 100,000 population. In the first graph the x-axis represents the estimated incidence per 100,000, while the y-axis represents the estimated mortality per 100,000 population calculated through the disease model. The second graph differs only by showing DALYs per case on the y-axis. The “Aggregate results” page also shows age-group and sex-stratified tables and bar charts.

**Additional file 5**. Colon surgery 2019 aggregated daly- BCoDE Toolkit output. Bubble charts are shown comparing different disease models, in which the size of each bubble corresponds to the magnitude of the burden of disease expressed in DALYs per 100,000 population. In the first graph the x-axis represents the estimated incidence per 100,000, while the y-axis represents the estimated mortality per 100,000 population calculated through the disease model. The second graph differs only by showing DALYs per case on the y-axis. The “Aggregate results” page also shows age-group and sex-stratified tables and bar charts.

**Additional file 6**. STROCSS CHECKLIST.

## Data Availability

The datasets used and/or analysed during the current study are available as Supplementary Material or from the corresponding author on reasonable request.
