# Supplementary material for: Hip prosthesis and colon surgery, a decade of surveillance on surgical site infections in Italy, a prospective cohort study: rates, trends, and disease burden in DALYs
Source: Antimicrob Resist Infect Control. 2024 Feb 12;13:17. doi: 10.1186/s13756-024-01377-6 (PMC10863245; doi:10.1186/s13756-024-01377-6)

Characteristics of Supplement Files.

As stated in the original publication of the ECDC BCoDE Toolkit (Colzani E, Cassini A, Lewandowski D, Mangen M-JJ, Plass D, McDonald SA, et al. (2017) A Software Tool for Estimation of Burden of Infectious Diseases in Europe Using Incidence-Based Disability Adjusted Life Years. PLoS ONE 12(1): e0170662. https://doi.org/10.1371/journal.pone.0170662) : the page “Aggregated results” compares the results of the selected disease models. Moreover, bubble charts are shown comparing different disease models, in which the size of each bubble corresponds to the magnitude of the burden of disease expressed in DALYs per 100,000 population. In the first graph the x-axis represents the estimated incidence per 100,000, while the y-axis represents the estimated mortality per 100,000 population calculated through the disease model. The second graph differs only by showing DALYs per case on the y-axis. The “Aggregate results” page also shows age-group and sex-stratified tables and bar charts.

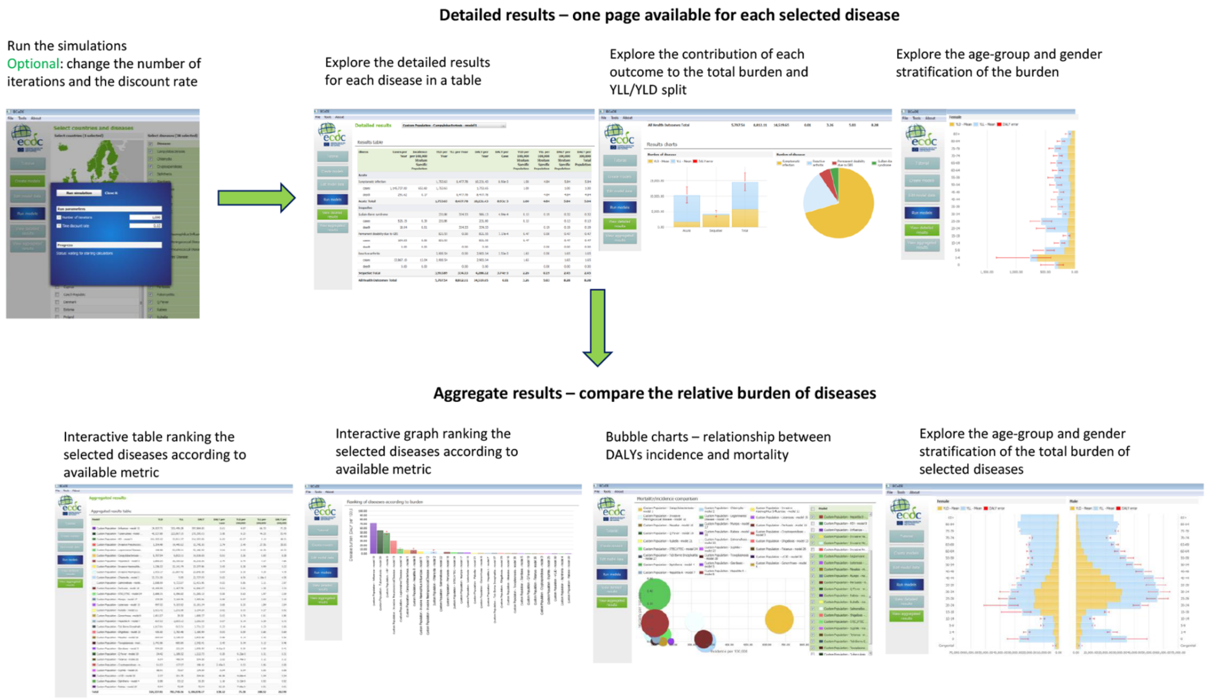

Supplement: Supplementary file 1 — Additional file 1. Characteristics of Supplement Files. [file 13756_2024_1377_MOESM1_ESM.pdf]
