# Supplementary material for: Hip prosthesis and colon surgery, a decade of surveillance on surgical site infections in Italy, a prospective cohort study: rates, trends, and disease burden in DALYs
Source: Antimicrob Resist Infect Control. 2024 Feb 12;13:17. doi: 10.1186/s13756-024-01377-6 (PMC10863245; doi:10.1186/s13756-024-01377-6)
Supplement: Supplementary file 2 — Additional file 2. Hip prosthesis 2019 aggregated daly - BCoDE Toolkit output. Bubble charts are shown comparing different disease models, in which the size of each bubble corresponds to the magnitude of the burden of disease expressed in DALYs per 100,000 population. In the first graph the x-axis represents the estimated incidence per 100,000, while the y-axis represents the estimated mortality per 100,000 population calculated through the disease model. The second graph differs only by showing DALYs per case on the y-axis. The “Aggregate results” page also shows age-group and sex-stratified tables and bar charts. [file 13756_2024_1377_MOESM2_ESM.pdf]

Aggregated results

PDF

Aggregated results table

Model	YLD	YLL	DALY	DALY per Case	YLD per 100,000	YLL per 100,000	DALY per 100,000	Cases per year	Incidence per 100,000	Deaths per year	Mor per 100
Custom Population - COLON SSI …	0.67	46.16	46.83	0.30	34.37	2,368.15	2,402.52	157.75	8,093.64	11.04	56
Custom Population - COLON SSI …	0.54	28.04	28.59	0.22	42.70	2,204.65	2,247.35	127.90	10,055.03	8.95	70
Custom Population - COLON SSI …	0.13	1.04	1.17	0.04	43.85	360.54	404.39	29.84	10,326.30	2.09	72
**Aggregate**	**1.34**	**75.24**	**76.58**	**0.24**	**38.17**	**2,143.60**	**2,181.77**	**315.49**	**8,988.26**	**22.08**	**62**

Ranking of diseases according to burden

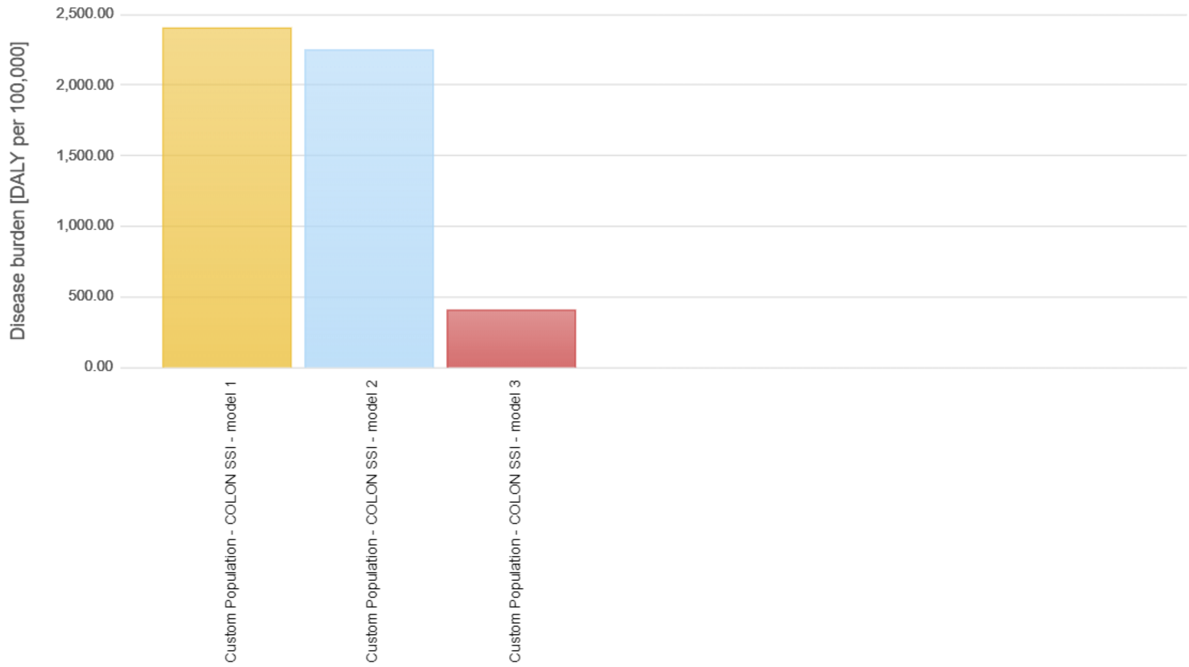

Mortality/incidence comparison

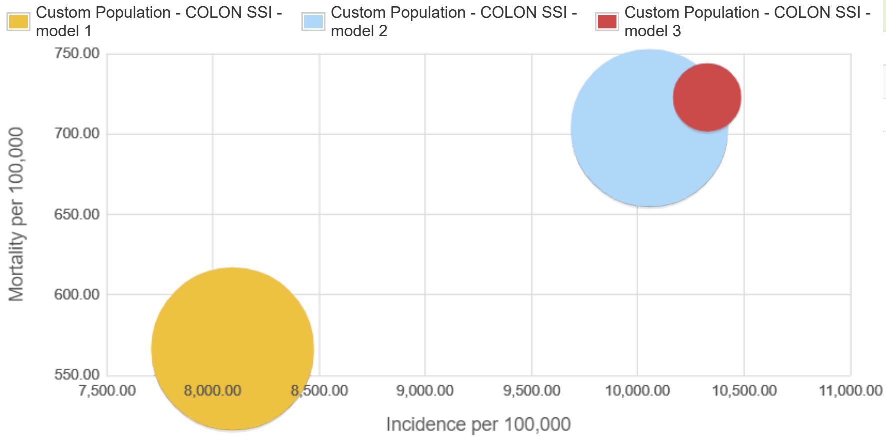

Model
Custom Population - COLON S…
Custom Population - COLON S…
Custom Population - COLON S…

DALY comparison

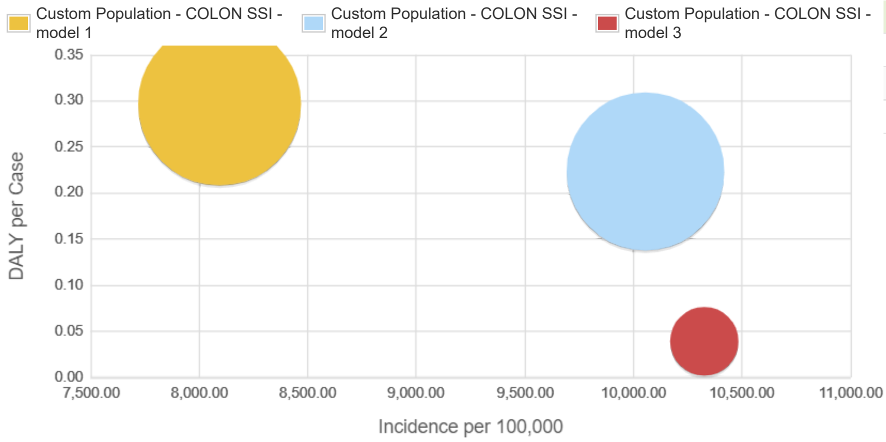

Model
Custom Population - COLON S…
Custom Population - COLON S…
Custom Population - COLON S…

Detailed results of total DALYs

1. DALY

Female

Age	2.5%	50%	97.5%	Mean
0	0.00	0.00	0.00	0.00
1-4	0.00	0.00	0.00	0.00
5-9	0.00	0.00	0.00	0.00
10-14	0.00	0.00	0.00	0.00
15-19	0.00	0.00	0.00	0.00
20-24	0.00	0.00	0.00	0.00
25-29	0.00	0.00	0.00	0.00
30-34	1.30	1.30	1.30	1.30
35-39	0.00	0.00	0.00	0.00
40-44	0.00	0.00	0.00	0.00
45-49	0.00	0.00	0.00	0.00
50-54	0.00	0.00	0.00	0.00
55-59	4.85	4.85	4.85	4.85
60-64	2.60	2.60	2.60	2.60
65-69	4.50	4.50	4.50	4.50
70-74	5.10	5.10	5.10	5.10
75-79	6.80	6.80	6.80	6.80
80-84	3.67	3.67	3.67	3.67
85+	1.91	1.91	1.91	1.91

Male

Age	2.5%	50%	97.5%	Mean
0	0.00	0.00	0.00	0.00
1-4	0.00	0.00	0.00	0.00
5-9	0.00	0.00	0.00	0.00
10-14	0.00	0.00	0.00	0.00
15-19	0.00	0.00	0.00	0.00
20-24	0.00	0.00	0.00	0.00
25-29	0.00	0.00	0.00	0.00
30-34	0.00	0.00	0.00	0.00
35-39	0.95	0.95	0.95	0.95
40-44	0.00	0.00	0.00	0.00
45-49	2.60	2.60	2.60	2.60
50-54	0.95	0.95	0.95	0.95
55-59	2.25	2.25	2.25	2.25
60-64	3.55	3.55	3.55	3.55
65-69	13.23	13.23	13.23	13.23
70-74	6.55	6.55	6.55	6.55
75-79	7.10	7.10	7.10	7.10
80-84	7.71	7.71	7.71	7.71
85+	0.95	0.95	0.95	0.95

Female **Male**

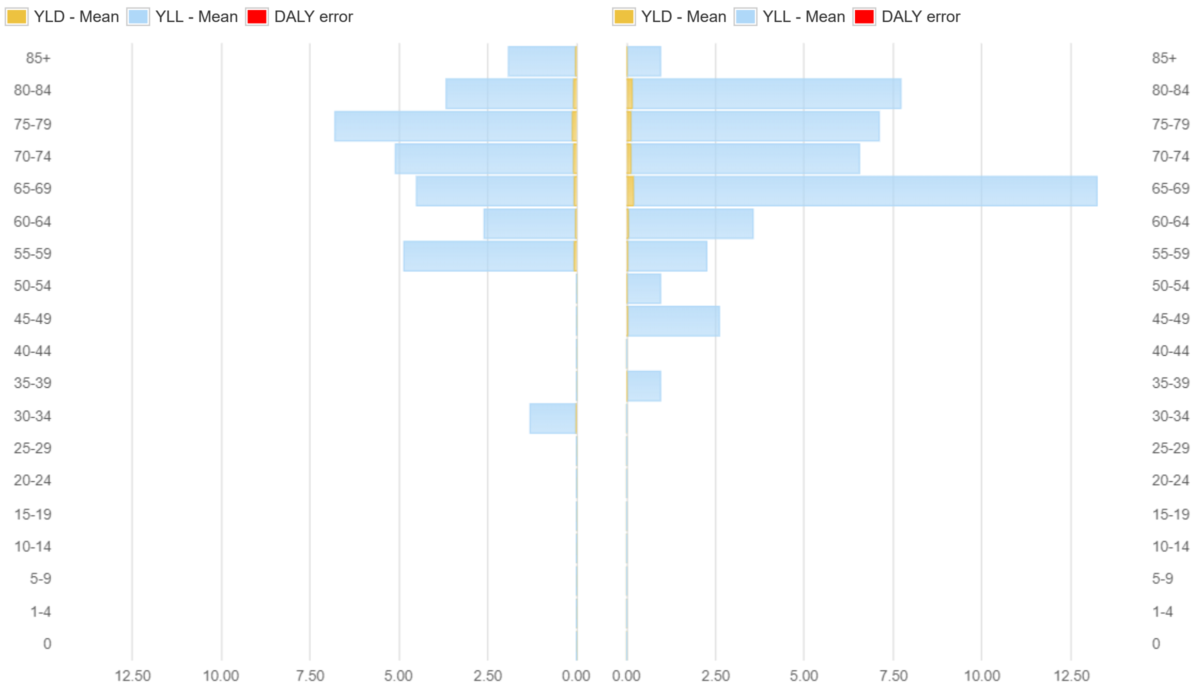

2. DALY per case

3. DALY per 100,000 Stratum Specific Population

4. DALY per 100,000 Total Population
